# Surgical outcomes of strabismus after iatrogenic ophthalmic artery occlusion caused by cosmetic filler injections

**DOI:** 10.1186/s12886-019-1264-1

**Published:** 2019-12-16

**Authors:** Hee Kyung Yang, Se Joon Woo, Seong-Joon Kim, Jeong-Min Hwang

**Affiliations:** 1 Department of Ophthalmology, Seoul National University College of Medicine, Seoul National University Bundang Hospital, 82, Gumi-ro 173 Beon-gil, Bundang-gu, Seongnam, Gyeonggi-do 13620 Republic of Korea; 2 Department of Ophthalmology, Seoul National University College of Medicine, Seoul National University Hospital, Seoul, Republic of Korea

## Abstract

**Background:**

To investigate the surgical outcomes of strabismus related to iatrogenic occlusion of the ophthalmic artery and its branches from cosmetic facial filler injection.

**Methods:**

A retrospective study was performed on 6 patients who underwent strabismus surgery among 23 patients who had suffered occlusion of the ophthalmic artery and its branches after cosmetic facial filler injection. Initial, preoperative and final ocular motility examinations, the type of surgery and surgical outcomes were evaluated.

**Results:**

At initial presentation, visual acuity was no light perception in 5 patients and hand motion in one patient. Five out of 6 patients showed initial ophthalmoplegia. Among these 5 patients, eye motility fully recovered in 3 patients although sensory strabismus developed during follow-up, while the remaining 2 patients had persistent ocular motility limitations. Strabismus surgery was performed at 2.2 ± 1.5 years after iatrogenic ophthalmic artery occlusion. Preoperatively, 5 of the 6 patients showed exotropia, and one patient had esotropia. Vertical deviation was found in 3 out of 6 patients in addition to the horizontal deviation. Successful outcome was achieved only in the 4 patients without persistent ophthalmoplegia after 1.4 ± 1.0 years from surgery. The other two patients with persistent ocular motility limitations failed to achieve successful alignment after surgery, and one patient eventually underwent evisceration due to phthisis bulbi.

**Conclusions:**

In our study, surgical outcomes of strabismus caused by cosmetic facial filler injection were successful only in patients without persistent ophthalmoplegia at the time of surgery.

## Background

Iatrogenic central retinal artery and/or ophthalmic artery occlusion could be caused by facial injection of filling materials [1, 2]. Visual loss is well known as a devastating complication of ophthalmic artery occlusion [1–3], whereas there is a lack of detailed description of the clinical course of strabismus and surgical outcomes related to this condition [4]. In a previous survey, ophthalmoplegia was initially present in 50% of patients with iatrogenic occlusion of the ophthalmic artery and its branches after cosmetic facial filler injections, and 33% had strabismus at the final examination [5].

To the best of our knowledge, there is no report considering outcomes of strabismus surgery after iatrogenic ophthalmic artery occlusion caused by cosmetic facial filler injections. Herein, we report the clinical characteristics and surgical outcomes of patients with strabismus related to iatrogenic ophthalmic artery occlusion.

## Methods

A retrospective review was performed of 23 consecutive patients who were referred to our hospital for central retinal artery occlusion and/or ophthalmic artery occlusion related to cosmetic facial filler injections between October 2008 and December 2017. Finally, 21 out of 23 patients who had sufficient information about their ocular motility examinations were included for analysis, and the clinical course of these patients were reported in a former study [4]. The study was approved by the institutional review board of Seoul National University Bundang Hospital and adhered to the tenets of the Declaration of Helsinki. Data were obtained from electronic medical records regarding age, sex, laterality of involved eye, injected filler material and injection sites, visual acuity, ocular motility examination, duration of follow-up and final sequelae. Final ocular motility was evaluated at least 3 months after onset. Therefore, two patients with a follow-up duration of less than 3 months were excluded, leaving 19 patients for final analysis.

Six patients had received strabismus surgery during follow-up. The type of surgery, intraoperative findings including forced duction test restults, and final surgical outcomes were noted. Ocular motility limitation of the involved muscle was graded on a scale of 1–4. The presence of strabismus and angle of deviation in the primary position was determined by the Hirschberg and Krimsky methods at near fixation. Sensory strabismus was defined as a secondary ocular deviation resulting from unilateral visual loss in the primary position and with no limitation in ductions and versions of the involved eye. Successful surgical outcome was determined when the near deviation in the primary position was between ≤10 prism diopters (PD) of exotropia and ≤ 5 PD of esotropia, and the vertical deviation was ≤5 PD.

## Results

Among the 21 patients with occlusion of the ophthalmic artery and its branches after cosmetic facial filler injections, initial ophthalmoplegia was found in 15 out of 21 patients (71%). On initial examination, the average number of involved muscles was 2.8 ± 1.2 (range, 1–4), and ophthalmoplegia involving all 4 recti muscles was found in 8 patients (38%). Ocular deviation in the primary position was orthotropic in 5 patients (33%), exotropia in 10 patients (67%), and none had esotropia. Five of the 10 patients with exotropia had concomitant vertical deviation. The mean angle of horizontal deviation in the primary position was 20.7 ± 15.8 PD (range, 0–40). Among the 5 patients with vertical deviation, 4 patients had hypertropia while one patient showed hypotropia, and the mean angle of vertical deviation was 14.0 ± 3.8 PD (range, 10–20).

At the final observation after an average follow-up of 29.8 ± 18.9 months, 11 out of 19 patients had strabismus; three patients with residual ophthalmoplegia and 8 patients with sensory exotropia. Strabismus surgery was performed purely for cosmetic purposes, and finally, 6 out of 11 subjects underwent strabismus surgery. Adjustable suture strabismus surgery was performed in all patients.

Among the 13 patients who had initial ophthalmoplegia and were followed-up for at least 3 months, ocular motility had fully recovered in 10 out of 13 patients (77%). The remaining three patients showed limitation of elevation (2 patients), abduction, depression and adduction by grades 1 to 3.

Sensory exotropia developed in 6 out of the 13 patients (46%) who had initial ophthalmoplegia. The mean angle of sensory exotropia was 25.8 ± 8.4 PD (range, 15–40) and one patient had concomitant hypertropia of 10 PD. Among the 6 patients with normal ocular motility at onset, 2 patients (33%) developed sensory exotropia with a deviation angle of 15 and 35 PD, respectively.

The clinical characteristics and treatment outcomes of the 6 subjects who underwent strabismus surgery are summarized in Table 1. Visual acuity was no light perception in 5 patients and hand motion in one patient. Surgery was performed at 2.2 ± 1.5 years after iatrogenic artery occlusion. Preoperatively, two patients had persistent ocular motility limitations due to incomplete recovery of ophthalmoplegia, and 4 patients had sensory exotropia with full ocular motility. Five of the 6 patients showed exotropia, and one remaining patient had esotropia. Three of the 6 patients showed vertical deviation in addition to the horizontal deviation.

**Table 1:** Clinical features of patients who underwent strabismus surgery after iatrogenic artery occlusion related to facial filler injection

No	Age	Laterality	Diagnosis	Injection substance	Injection site	Initial duction	Time from onset to surgery (y)	Preoperative duction	Preoperative deviation	Type of surgery^†^	Follow-up (y)	Final outcome
1	39	R	OAO	AF	glabella	full	4.3	full	XT 35PD	LR Rc 6 mm, MR Rs 4.5 mm	0.8	Ø
2	27	R	OAO	HA	nasal dorsum, glabella	adduction −3, depression − 3	0.6	full	XT 20PD	LR Rc 8 mm, MR Rs 5.5 mm	0.2	Ø
3	31	L	CRAO	HA	nasal dorsum	abduction −3, adduction −1, elevation − 1, depression − 3	1.0	full	XT 40PD, HT 16PD	LR Rc 8 mm, MR Rs 5 mm with infratransposition	1.6	Ø
4	19	L	CRAO	AF	glabella	adduction −1	2.9	full	XT 40PD	1st: Enophthalmos correction with subperiosteal wedge implant	3.0	Ø
										2nd: LR Rc 10 mm, MR Rs 8 mm		
5	24	R	OAO	HA	nasal dorsum	abduction −3, adduction −3, elevation − 3, depression − 3	3.1	elevation −1	XT 30PD, HoT 5PD, R DVD	LR Rc 6.5 mm, MR plication 5.5 mm, SR Rc 5 mm	0.6	ET 5PD, HoT 16PD
6*	30	R	CRAO	HA	nasal dorsum	abduction −3, adduction −4, elevation − 3, depression − 3	0.5	abduction − 3, depression −1	ET 18PD, HT 10PD	MR Rc 5 mm, SR Rc 3 mm	3.7	ET 6PD, HT 20PD

Abbreviations: y, years; R, right; L, left; OAO, ophthalmic artery occlusion; CRAO, central retinal artery occlusion; AF, autologous fat; HA, hyaluronic acid; XT, exotropia; PD, prism diopter; ET, esotropia; HT, hypertropia; HoT, hypotropia; DVD, dissociated vertical deviation; Ø, orthotropia, LR, lateral rectus; MR, medial rectus; Rc, recession; Rs, resection

*Case 6 eventually underwent evisceration with Medpor implant due to phithis bulbi. ^†^Adjustable suture strabismus surgery was performed in all cases

Regarding the type of surgery, lateral rectus recession and medial rectus resection was performed in the 4 patients with sensory exotropia and full ocular motility. Intraoperative forced duction tests revealed no definite restriction of the extraocular muscles in these patients, except for a mild restriction of the left lateral rectus muscle in Case 4. All four patients showed good motor outcomes of orthotropia at the final examination.

Two patients had residual ophthalmoplegia at the time of strabismus surgery. Case 5 had exotropia, a small angle of hypotropia and large angle of dissociated vertical deviation. Lateral rectus recession, medial rectus plication and superior rectus recession was performed, which resulted in consecutive esotropia and worsened hypotropia. In another patient with esotropia and hypertropia (case 6), medial rectus recession and superior rectus recession resulted in residual esotropia and hypertropia. Intraoperative findings in Case 5 and 6 revealed atrophy and fibrosis of the involved muscles with mild restriction.

Successful outcome was achieved only in the 4 patients without any residual ophthalmoplegia after 1.4 ± 1.0 years from surgery. The other two patients with persistent ocular motility limitations failed to achieve successful alignment after surgery, and one patient eventually underwent evisceration due to phthisis bulbi.

## Discussion

To the best of our knowledge, there is no report considering outcomes of strabismus surgery after iatrogenic occlusion of the ophthalmic artery and its branches caused by cosmetic facial filler injections. Up to now, little is known of the clinical course and surgical outcomes of ophthalmoplegia and sensory strabismus related to iatrogenic artery occlusion after cosmetic facial filler injection [3, 4]. In this study, we found that strabismus surgery was successful only in patients without residual ophthalmoplegia.

The ophthalmic artery is the first branch of the internal carotid artery. Its branches supply all orbital structures and also part of the nose and face. Occlusion of the ophthalmic artery or its branches can produce sight-threatening conditions. Arterial supply of the extraocular muscles originate from the inferior branch of the ophthalmic artery [6, 7], therefore, ophthalmic artery occlusion by cosmetic facial fillers could impair extraocular muscle function, resulting in ophthalmoplegia.

In our case series of ophthalmoplegia after cosmetic facial filler injection, ocular motility fully recovered in 77% of patients [4]. The remaining three patients showed limitation of elevation (2 patients), abduction (1 patient), depression (1 patient) and adduction (1 patient) by grades 1–3. Zimmerman et al. [8] reported that ophthalmoplegia improved in most patients with orbital infarction after neurosurgical procedures. Among those with normal ocular motility at initial presentation, 33% of patients developed sensory exotropia at the final examination. Consequently, 6 patients received strabismus surgery, and successful outcome was achieved only in the 4 cases with normal ocular motility at the time of surgery. Abnormal muscle functions may reduce the predictability of strabismus surgery, which is caused by structural alterations of the extraocular muscles due to ischemic injury and denervation. Diffuse muscle atrophy and fibrosis observed in the patients with persistent ophthalmoplegia (Case 5 and 6) supports this notion.

Our study has some limitations. First, this is a retrospective study. Follow-up schedules were variable and overall short. Second, only a small number of patients were included, even though this is the largest series regarding the surgical results of sensory strabismus and ophthalmoplegia after cosmetic facial filler injection. That is why the standard deviations for all parameters are relatively high suggesting large variability. Therefore, our results cannot be generalized to all such cases.

## Conclusion

In our study, strabismus surgery was successfully performed in patients with sensory exotropia caused by cosmetic facial filler injections, provided that ocular motility was normal at the time of surgery. In contrast, patients with persistent ophthalmoplegia failed to achieve successful alignment after surgery probably due to structural alterations of the injured muscles.

## Data Availability

Data for this case report were collected by chart review of the patient’s electronic medical record, which is not publicly available due to privacy considerations.

## Abbreviations

PD: prism diopters
